# Development and Psychometric Evaluation of a Japanese Version of Newly Graduated Nurses’ Difficulties with End-of-Life Care for Cancer Patients (NDEC Scale)

**DOI:** 10.3390/nursrep12030063

**Published:** 2022-09-01

**Authors:** Akitoshi Asano, Sayuri Sakai, Nao Seki, Yu Koyama

**Affiliations:** 1 Graduate School of Health Sciences, Department of Nursing, Doctoral Program, Niigata University, 2-746 Asahimachi-dori, Chuo-ku, Niigata 951-8518, Japan; 2 Graduate School of Health Sciences, Niigata University, 2-746 Asahimachi-dori, Chuo-ku, Niigata 951-8518, Japan

**Keywords:** end-of-life care, reliability and validity, nurse, oncology nursing

## Abstract

(1) Background: End-of-life care (EoL care) for cancer patients is stressful for nurses and can easily lead to burnout. Newly graduated nurses (NGNs) have a particularly difficult time, but no scale or inventory has been designed to evaluate their difficulties. This study developed and tested the reliability and validity of a scale to measure NGNs’ difficulties with EoL care for cancer patients (NDEC scale). (2) Methods: This study population consisted of 1000 NGNs and 1000 nurses with at least five years of clinical experience (GNs) that were working in hospitals in Japan. The initial scale consisted of six factors and 28 items. The reliability and validity of the scale were tested. (3) Results: A total of 171 NGNs and 194 GNs responded to the survey. The scale consisted of five factors and 25 items with the factors including “Feeling painful”, “Can’t deal with patients and their families”, “Don’t know the answer”, “Cannot afford”, and “Being afraid of death”. The criteria validity, known population validity, and internal consistency were confirmed. (4) Conclusions: The scale was validated to have a certain level of reliability and validity. The NDEC scale is expected to be used for self-care for NGNs and as an effectiveness indicator for educational programs.

## 1. Introduction

It is feared that nursing cancer patients in the period just before death cause a great deal of stress to nurses themselves, leading to turnover and burnout [1]. Previous studies on the difficulties in cancer nursing have found that a lack of knowledge, skills, and communication with physicians and medical staff [2] is difficult. In particular, newly graduated nurses (NGNs) are typically working under stressful conditions, as they lack the necessary knowledge and skills as nurses, and perform their daily duties with anxiety concerning communication with patients, their families, and their colleagues [3,4,5]. In previous studies, NGNs who had EoL care experiences with cancer patients were reported to have negative emotions, such as shock about death, and these experiences have been reported to increase employee turnover [6,7,8,9]. As compared to noncancer patients, cancer patients are characterized by a rapid deterioration in their condition after the first month of prognosis. They are prone to physical and mental suffering within a short period of time [10] that may require specialized knowledge and skills for adequate care. In the future, as Japan’s population continues to age and the expected number of terminal cancer patients is expected to increase [11], there could be a need for the further enhancement of EoL care. This could also assist NGNs, and the need for education on EoL care is clearly stated in the “Training Guidelines for New Nursing Staff,” established by the Ministry of Health, Labor, and Welfare in Japan. However, while it has been conducted as part of group training for NGNs and education specific to EoL care, effectiveness indicators have not been established. Previous studies [7,8] have shown that NGNs with little nursing experience may feel a reasonable burden when they undertake EoL care experience. In particular, NGNs have difficulty communicating with dying patients and their families, and need technical as well as emotional support [8].

Several scales to measure the nurses’ difficulties with providing EoL care for cancer patients and their families have been developed [2,12,13]; these scales have been used as effectiveness indicators for educational programs. EoL care is a new experience for NGNs with variables factors and needs. Considering the psychological burden on NGNs when faced with patient suffering, supportive training that is more than instruction in knowledge and skills is necessary. The characteristics of NGNs’ difficulties are their painful feelings and difficult experiences, such as fear of death, regret, and helplessness, and include ethical problems as well as conflicts [7,9]. Therefore, it is necessary to provide support that focuses on the experiences of NGNs, rather than simply recognizing the causes of their difficulty or teaching them knowledge and skills. Therefore, we developed a difficulty scale that would allow NGNs to identify their own difficult experiences, share their difficult experiences with senior nurses, and provide an opportunity to receive support from senior nurses. This study would not only reduce Japanese NGNs’ difficulties, ethical problems, and conflicts, but could also be used as an effectiveness indicator for educational programs. The purpose of this study was to develop and to test the reliability as well as validity of a scale to measure NGNs’ difficulties with EoL care for cancer patients (the NDEC scale).

## 2. Materials and Methods

This study was conducted in three phases: (1) selecting items, (2) examining the content validity of the items of the NDEC scale in addition to refining expressions, and (3) testing its reliability and validity. 

### 2.1. Phase 1: Selecting Items

We defined the negative emotions that NGNs feel, such as anxiety, fear, shock, conflict, lack of confidence, doubt, sadness, pain, and regret, when they experience difficulties in the EoL care for cancer patients. The first step was a literature review of NGNs’ difficulties in EoL care. The terms “end-of-life care”, “newly graduated nurse”, and “difficulty” were used to search for relevant literature on the Ichushi Web (The Japan Medical Abstract Society). Results indicative of NGNs’ difficulties were extracted and coded. Codes thought to reflect similar semantic content were grouped into subcategories, categories, and core categories. Fifty-five subcategories, which were appropriate as abstractions suitable for use as items, were adopted as the items for query. We sent 55 items to NGNs working at cancer treatment hub hospitals in Japan (the response rate was 22%, 101/449). The results were tabulated, factor analysis was conducted, and 21 items were selected. The free descriptions of the 37 respondents of NGNs’ difficulties were collected, as were the 55 items, and were analyzed using the qualitative synthesis method (KJ method). These free descriptions data were carefully read and led to the creation of 90 original labels, in which 7 levels of grouping and 6 symbols were found: crying, fear of end-of-life care, helplessness, dilemma, senior nurse, and motivation for learning. Six symbols were selected for items; the total number of the NDEC scale was 27 items. 

### 2.2. Phase 2: Examining the Content Validity of the Items of the NDEC Scale and Refining Expressions

A preliminary draft was tested through a survey of NGNs working across 100 hospitals in Japan [14]. The authors shortlisted 10 blocks from a list published by the Japan Hospital Association, and 10 hospitals were randomly selected from each block. The inclusion criteria for NGNs were as follows: (1) completion of basic nursing education in March 2020, and (2) having no clinical experience with other facilities. The exclusion criteria were as follows: (1) no experience in caring for cancer patients and (2) working in a department other than the general ward. An online survey was conducted through a research company in Japan between October 2020 and December 2020. Regarding previous studies [15,16], descriptive statistics of the items were calculated, and items with biased responses or similar semantic content were modified or deleted. First, the mean and standard deviation (SD) of each item were calculated. Second, the selection criteria were set as follows: floor effects (FEs)/ceiling effects (CEs): 1 less or 7 over (mean ± SD); item-total point correlation (IT-C): rs < 0.3; and inter-item correlation coefficient (IICC): rs > 0.7. Items meeting these criteria were considered for modification or deletion. Three NGNs (excluding the participants) with experience in EoL care for cancer patients, one oncology clinical nursing specialist (OCNS) with experience in palliative care, one university academic member with expertise in cancer nursing, and the researcher conducted discussions to assess the content validity of the items. As a result, 50 NGNs participated in the study, and 9 items met the criteria. Discussions with the NGNs, OCNS, academic member, and the researcher resulted in some remarks regarding the expression of the items, and some items contained two pieces of semantic contents in one item. Based on these discussions, item expressions were modified and inappropriate items were deleted, and the NDEC scale consisted of 28 items.

### 2.3. Phase 3: Testing Reliability and Validity

#### 2.3.1. Participants and Procedure

We stratified the 10 blocks from a list published by the Japan Hospital Association [17] and randomly selected 100 hospitals in each block; 1000 general hospitals in Japan were randomly selected. We selected one NGN and one general nurse (GN) from each hospital. The inclusion criteria were as follows: (1) NGNs were those with less than 1 year of post-qualification experience when first employed at the hospital. They graduated from nursing school in March 2020 and obtained employment in April 2020. (2) GNs were based on Benner’s definition [18], with at least five years of clinical experience, including certified nurses (CNs) and clinical nurse specialists (CNS). The exclusion criteria were as follows: (1) over 30 years old (only NGNs), (2) no experience of caring for cancer patients, and (3) working in a department other than the general ward. In February 2021, one copy of each of the research request documents and envelopes to be distributed to NGNs and GNs were sent to the head nurses of the selected facilities. The envelope contained a research request document, a paper questionnaire, and a leaflet with a URL to answer the questionnaire via the Internet. Participants were given a choice to complete the paper questionnaire or the leaflet. For the Internet survey, we used an Internet survey system provided by a Japanese research company [19]. The data collection period was from February 2021 to March 2021. We did not send any reminders or provide any incentives to solicit the participants in this study.

#### 2.3.2. Survey Items

The survey included the following demographic queries: sex, age, advisor, the NDEC scale’s 28 items, and a numerical rating scale to measure NGNs’ difficulties (the NRS). The responses to the NDEC scale could be provided via a 7-point Likert-type scale (“strongly agree”, “agree”, “somewhat agree”, “neither”, “somewhat disagree”, “disagree”, and “strongly disagree”), with higher scores indicating a stronger difficulty. Reverse scoring was not used. Items were prepared in Japanese. The NRS rated the perceived difficulty of experiencing EoL care on a scale of 0 to 10, with higher numbers indicating greater difficulty.

#### 2.3.3. Data Analysis

A descriptive statistical analysis was conducted. Reliability was assessed via internal consistency. We conducted an exploratory factor analysis with Promax rotation and maximum likelihood; standard regression coefficients were taken as the minimum of 0.4, and Cronbach’s coefficient alpha for each factor was evaluated. Validity was assessed via construct validity, criterion validity, and known-group validity. Construct validity was assessed using exploratory and confirmatory factor analyses. The exploratory factor analysis was conducted to extract constructs of the NDEC scale, and the confirmatory factor analysis was conducted to consider the NDEC scale model, using the results of the exploratory factor analysis. The criteria validity analysis was assessed via the correlation between the NDEC scale total score and the NRS, the score of each factor, and the NRS. Spearman’s correlation coefficient was used for the analysis. Known-group validity was assessed by the relationship between the NDEC scale total scores of the NGNs and GNs. We also assessed the relationship between the score of each factor of the NGNs and GNs. The Mann–Whitney U-test was used for analysis. Statistical analyses were performed using the Statistical Package for Social Sciences (SPSS) (version 25, Armonk, NY, USA) and Analysis of Moment Structure (AMOS) (version 20, Armonk, NY, USA). Statistical significance was set at the *p* < 0.05 level. Methodological quality appraisal was performed with reference to the recommendations of consensus-based standards for the selection of health measurement instruments (COSMIN) [20].

#### 2.3.4. Ethical Approval

This research was approved by the ethical review committee of the affiliated university (Approval No. 2020-0169). The researcher provided explanations for the participants about the study’s purpose and methods, the voluntary nature of research cooperation, data storage, the protection of personal information, and the publication of the research results in the research request documents enclosed in the envelope. The participants were informed that there were no professional disadvantages in declining participation; they could discontinue whenever they felt uncomfortable or had difficulties while answering; and that anonymity would be preserved. Consent of participation was assumed if the submit button on the website was clicked or if a paper-based questionnaire was returned. Paper questionnaires were returned to the corresponding author’s affiliation and stored in a locked safe at the university. The Internet survey responses were collected by the research company, and the results of the collection were reviewed by accessing a dedicated “My Page”. The ID and password required to access the secure results were managed by the corresponding author. The research company has an SSL/TLS-encrypted communication system and discloses its privacy and information security policies on its website.

## 3. Results

The NDEC scale was created in Japanese. For the submission of this paper, the questionnaire and the results of the exploratory factor analysis were proofread by native speakers and translated from Japanese into English.

### 3.1. Responses

Table 1 summarizes the characteristics of the participants. Of the 1000 NGNs (total of the paper and web surveys), 171 (male: 5, female: 166) were included (response rate: 18%); for GNs, 194 (male: 21, female: 173) were included (response rate: 19%). The average age of the NGNs was 23.00 ± 2.32, and for GNs it was 34.08 ± 8.30. The NRS was 7.22 ± 1.46 for NGNs, and 6.54 ± 2.01 for GNs. All of the respondents indicated that they had advisors.

### 3.2. The NDEC Scale’s Reliability and Validity

#### 3.2.1. Reliability of the NDEC Scale

Table 2 shows the internal consistency results. Cronbach’s alpha for each domain ranged from 0.72 to 0.83, and the entire scale was 0.90.

#### 3.2.2. Validity of the NDEC Scale

**Figure 1:**
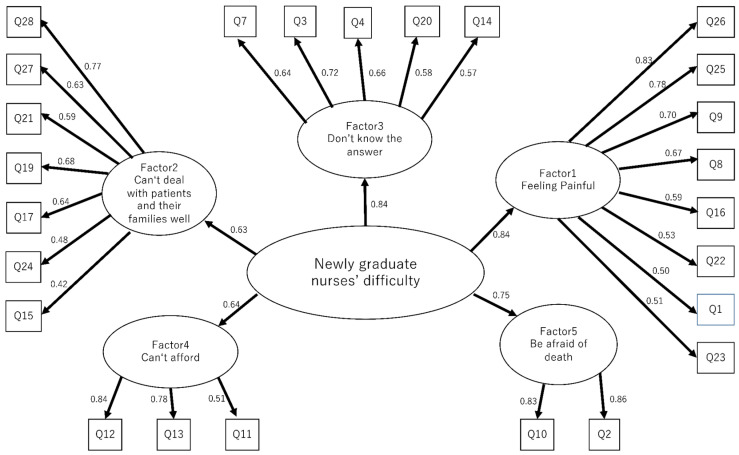
Results of the confirmative factor analysis. Note: The model fitness index was X^2^ = 632 (*p* < 0.001, degree of freedom = 270), GFI = 0.770, AGFI = 0.720, CFI = 0.790, and RMSEA = 0.080.

Table 2 shows the results of the exploratory factor analysis. We completed the NDEC scale with five domains and 25 items. Each domain was titled: Factor 1, “Feeling Painful”; Factor 2, “Can’t deal with patients and their families well”; Factor 3, “Don’t know the answer”; Factor 4, “Can’t afford”; and Factor 5, “Being afraid of death”. Using the factor structure identified as a result of the exploratory factor analysis, a confirmatory factor analysis was conducted (Figure 1). The model adopted was a higher-order factor model, in which the five factors extracted as latent variables of the questionnaire items, which were observational variables, were primary variables, and the secondary factor, which was a higher-order factor explaining the primary variable, was difficult. The model fitness index for the NDEC scale was X^2^ = 632, *p* < 0.001, degree of freedom = 270, GFI = 0.770, AGFI = 0.720, CFI = 0.790, and RMSEA = 0.080. Table 3 shows the results of criteria validity, confirmed by the correlation between the NDEC scale total score and the NRS, with a correlation coefficient of 0.43. The score of each factor and the NRS ranged from 0.25 to 0.40. Table 4 shows the result of known-group validity; we confirmed a significant difference between the NDEC scale total scores of the NGNs and GNs as well as the scores of each factor.

## 4. Discussion

### 4.1. The Difficulties of NGNs in EoL Care

In a previous study of NGNs [7,21], it was reported that NGNs were prone to the difficulty with EoL care, but as the study was based on individual interviews, the actual situation was not clarified. This study identified factors of difficulties, namely “Feeling Painful”, “Can’t deal with patients and their families well”, “Don’t know the answer”, “Can’t afford”, and “Being afraid of death”. The latent factors of the difficulty were different from those involving a lack of knowledge or skills as well as multidisciplinary cooperation that were revealed in previous surveys of general nurses [3,13]. The difficulties for NGNs involved fears, dilemmas, and conflicts that were characteristic of NGNs.

### 4.2. Reliability and Validity of the NDEC Scale

The internal consistency of the scale was confirmed by Cronbach’s alpha coefficient, which met the desirable criterion of 0.7 or higher [22]. Content validity was confirmed. For construct validity, a factor structure consisting of 25 items of five factors was extracted as a result of an exploratory factor analysis, and a confirmatory factor analysis was conducted using the results. The model created was a higher-order factor model located from the second-order factors to the five latent variables, which were the first-order factors, and from the first-order factors to the observed variables, which were capable of explaining the specific difficulty from the higher-order factors. Furthermore, the latent variables for the difficulties identified in this study reflected the results of previous studies [6,7,9]. Although the model fitness index was low, the model itself was considered to be a clinically useful result and had a certain degree of construct validity. In Japan, opportunities to experience the dying process have been decreasing, owing to the shift to nuclear families and changes in the place of death [11]. Experiencing EoL care for cancer patients is a close and personal experience of the dying process, which is an unknown experience for NGNs [23,24]. Therefore, they are likely to experience fear of death and painful emotions. It is easy to feel fear of death and painful emotions [25]. The result of the criteria validity was compared with the NRS, which is a subjective indicator of these difficulties. The results of known-population validity indicated that the questions in the scale were specific to NGNs. Therefore, in this study, the difficulty scale was considered to have a certain degree of validity in measuring the difficulties specific to NGNs.

### 4.3. Availability of the NDEC Scale

The presence of a senior nurse, who could be consulted at any time when difficulties arose, was considered important, and it has been emphasized that in facilities without such a nurse, NGNs were unable to consult anyone, which could lead to burnout and increase employee turnover [26,27]. EoL care is a stressful experience for NGNs, but it is also a valuable opportunity for them to grow as nurses. NGNs need the support of GNs to help them express their feelings and reflect on their nursing care [28]. The importance of GNs has been indicated in guidelines for the education of NGNs, not only in EoL care [29,30]. The NDEC scale embodied NGNs’ difficulties, and sharing the completed NDEC scale with GNs could provide an opportunity to receive support. NGNs who receive support could be more motivated to learn EoL care for patients and families, leading to their growth as nurses. Since approximately half of NGNs have been estimated to be in a state of burnout, and EoL care is one of the factors contributing to burnout, the use of the NDEC scale could allow them to receive support from GNs, which could help to reduce burnout [1,31]. In addition, the use of the NDEC scale could increase motivation to learn about EoL care and improve EoL care. We considered the idea that the NDEC scale could be used as an effectiveness indicator for educational outcomes. Additional research using a burnout scale should be conducted to confirm the effectiveness of the NDEC scale.

### 4.4. Limitations

Firstly, this study was not tested for a test–retest study. Specific sampling and analysis methods should be considered in the future to further verify its reliability. Secondly, this study was based on a literature review and a questionnaire survey of difficulties in Japan. In the future, linguistic validity between Japanese and English should be verified. Thirdly, this study had a low response rate. The reasons for this included the following: (1) the contents of this survey reminded NGNs of stressful situations of EoL care; (2) the survey period coincided with the spread of COVID-19 in Japan. It is necessary to accumulate data through the continued use of the NDEC scale as an indicator of educational outcomes and to reexamine a confirmatory factor analysis and model fit index in the future.

## 5. Conclusions

The NDEC scale consisted of five factors and 25 items: “Feeling Painful”, “Can’t deal with patients and their families well”, “Don’t know the answer”, “Can’t afford”, and “Being afraid of death”. Reliability and validity were thus confirmed. Using the NDEC scale, NGNs will be able to specify their difficulties and receive support from GNs. Furthermore, it may be used as an indicator for educational programs and to contribute to the education of EoL care for NGNs. In the future, we will continue using the NDEC scale to conduct additional surveys simultaneously while using the burnout scale, and we will accumulate data as an indicator for educational programs.

## Figures and Tables

**Table 1:** Characteristic of this study’s participant’s.

NGNs = 171, GNs = 194			
NGNs’ *n* (%) or Mean ± SD		GNs’ *n* (%) or Mean ± SD	
Male	5 (3)		21 (11)
Female	166 (97)		173 (89)
Age (Years)	23.00 ± 2.32		34.08 ± 8.30
NRS	7.22 ± 1.46		6.54 ± 2.01
CN or CNS license	None		41 (21)
Advisors (multiple selection)	171 (100)		194 (100)
Individual educator (ex preceptor)	144	Same department nurse	178
Senior nurses other than individual educator	143	Same hospital CN or CNS	112
New graduate nurse in the same hospital	93		
Head nurse	45	Head nurse	113
Nursing school classmate	53	Nursing school classmate	27
Family	37	Family	25
No advisor	0	No advisor	0
Others	0	Others	11
		Doctor	5
		Another hospital CN	4
		Another hospital CNS	1
		Pharmacist	1

NGNs: newly graduate nurses, GNs: general nurses, NRS: numeric rating scale to measure the NGN’s difficulties, CN: certified nurse, and CNS: clinical nurse specialist.

**Table 2:** Results of the exploratory factor analysis.

	Standard Regression Coefficients				
	Factor 1	Factor 2	Factor 3	Factor 4	Factor 5
**Factor1: Feeling Painful** α = 0.83 mean ± SD = 4.57 ± 0.98					
Q26 I was sad about the care just before the patient died	0.84	0.34	0.46	0.34	0.42
Q25 I felt empty in the care just before the patient died	0.81	0.38	0.48	0.41	0.31
Q9 I couldn’t accept that the patient died	0.69	0.22	0.43	0.37	0.41
Q8 Conflict with having to prepare for bereavement while the patient is still alive	0.67	0.22	0.40	0.26	0.35
Q16 After experiencing patients’ death several times, I felt painful and couldn’t see the surroundings	0.58	0.24	0.23	0.32	0.50
Q22 I thought I shouldn’t ask the patient about death	0.51	0.37	0.20	0.41	0.38
Q1 I’m confused as to whether it’s good or bad to cry when a patient dies	0.49	0.08	0.41	0.25	0.40
Q23 I hesitated to talk to the patient about the last moment, thinking that it would be depressing	0.48	0.41	0.28	0.43	0.28
**Factor2: Can’t deal with patients and their families well** α = 0.78 mean ± SD = 5.30 ± 0.81					
Q28 I felt that I was an inconvenience to the patient due to my lack of knowledge and skills	0.32	0.81	0.36	0.42	0.03
Q27 I was just doing what my seniors told me, and I couldn’t predict the medical condition	0.22	0.67	0.12	0.32	0.22
Q21 I couldn’t afford to grasp the patient’s condition because I was busy with work	0.15	0.64	0.15	0.26	0.03
Q19 I regret not being able to support the patient	0.50	0.63	0.59	0.54	0.15
Q17 feel that my ability to assess patients is weak	0.23	0.63	0.35	0.52	0.26
Q24 I was having trouble dealing with a family member who did not express emotions	0.35	0.47	0.27	0.35	0.15
Q15 Difficult to communicate with the family of patients with reduced consciousness	0.33	0.40	0.30	0.34	0.18
**Factor3: Don’t know the answer** α = 0.75 mean ± SD = 5.65 ± 0.83					
Q7 I want to learn to become a nurse who meets the needs of patients and their families	0.33	0.25	0.73	0.31	0.25
Q3 No matter how many times I experience final hours care, I feel uncomfortable	0.58	0.21	0.67	0.27	0.46
Q4 Final hours care is always an unanswered question	0.39	0.23	0.66	0.21	0.27
Q20 I was wondering if this was all right for the care of the patient just before patient died	0.46	0.57	0.61	0.44	0.11
Q14 Difficult to deal with patients who cannot make decisions	0.43	0.44	0.47	0.43	0.25
**Factor4: Can’t afford** α = 0.72 mean ± SD = 5.31 ± 0.98					
Q12 When the patient asked me about my condition, I was very upset	0.37	0.47	0.35	0.98	0.22
Q13 I was worried when the patient asked me about the prognosis	0.46	0.46	0.30	0.71	0.30
Q11 The patient suddenly changed and I was upset	0.34	0.46	0.30	0.51	0.33
**Factor5:Being afraid of death** α = 0.82 mean ± SD = 5.01 ± 1.38					
Q10 There is a vague fear of death	0.55	0.24	0.43	0.34	0.85
Q2 Somewhere there is a fear of facing death	0.56	0.28	0.45	0.35	0.76
Cumulative Contribution ratio: 50%. Cronbach’s α of the entire scale: α = 0.9.					
α: Cronbach’s coefficient alpha. SD: standard deviation					
Highlighted areas indicate standard regression coefficients of 0.4 or higher.					

**Table 3:** Criterion validity of the NDEC scale.

	Factor 1	Factor 2	Factor 3	Factor 4	Factor 5	Total
NRS	0.34 **	0.30 **	0.39 **	0.25 **	0.40 **	0.43 **

NDEC scale: a scale to measure NGNs’ difficulties with end-of-life care for cancer patients. ** *p* < 0.01. NRS: numeric rating scale to measure the NGNs’ difficulties. Spearman’s correlation coefficient was used for the correlations between the NDEC scale total score and the NRS, the score of each factor, and the NRS.

**Table 4:** Known-group validity of the NDEC scale.

	NGNs (*n* = 171)		GNs (*n* = 194)		
	Mean	SD	Mean	SD	*p*-Value
Total	142.81	19.42	118.05	27.91	<0.01 *
Factor 1	4.56	1.01	3.71	1.08	<0.01 *
Factor 2	5.30	0.82	4.18	1.07	<0.01 *
Factor 3	5.66	0.84	5.45	0.90	0.03 *
Factor 4	5.30	1.03	4.16	1.37	<0.01 *
Factor 5	5.01	1.41	3.50	1.47	<0.01 *

SD: standard deviation. NGNs: newly graduate nurses. GNs: general nurses. * *p* < 0.05. NDEC scale: a scale to measure NGNs’ difficulties with end-of-life care for cancer patients. The statistical analysis carried out with the Mann–Whitney U test.

## Data Availability

The data used in this study are not open to other researchers at this time.
